# Syndrome de Miller Fisher avec anticorps anti GQ1b négatif au cours d'une pneumonie à *Mycoplasma pneumoniae*

**DOI:** 10.11604/pamj.2013.15.122.2750

**Published:** 2013-08-07

**Authors:** Victor Sini, Calixte Kuate Tegueu, Séraphin Nguefack, Mathieu Boone, Richard Roos-Weil

**Affiliations:** 1 Faculté de Médecine et des Sciences Biomédicales, Université de Yaoundé 1, Cameroun; 2 Centre Hospitalier Universitaire Amiens Nord, France; 3 Centre Hospitalier de Compiègne, France

**Keywords:** Syndrome de Miller Fisher, Mycoplasma pneumoniae, ganglioside GQ1b, Miller Fisher syndrome, Mycoplasma pneumoniae, ganglioside GQ1b

## Abstract

Le Syndrome de Miller Fisher est caractérisé par l'association d'une ophtalmoplégie, d'une ataxie et d'une aréflexie ostéo-tendineuse. Une infection virale est le plus souvent retrouvée dans les jours ou semaines qui précèdent la symptomatologie. Nous rapportons un cas de syndrome de Miller Fisher survenu chez une femme de 75 ans, et ce au décours d'une infection pulmonaire à *Mycoplasma pneumoniae*. Les sérologies virales habituelles étaient négatives. Les anticorps anti GQ1b étaient absents. Il n'y avait pas de lésion du tronc cérébral à l'imagerie par résonnance magnétique. L’évolution clinique était favorable après perfusion d'immunoglobulines humaines polyvalentes et des macrolides en comprimés. La sérologie *Mycoplasma pneumoniae* doit être systématiquement recherchée dans le bilan du syndrome de Miller Fisher.

## Introduction

Le syndrome de Miller Fisher est une affection démyélinisante aigue du système nerveux périphérique, décrit pour la première fois par Fisher en 1956 [1]. Il s'agit d'une affection associant classiquement une ataxie, une ophtalmoplégie et une aréflexie ostéo-tendineuse généralisée. Il a été décrit comme une forme clinique du syndrome de Guillain Barré avec lequel il partage plusieurs similitudes. Une infection virale est le plus souvent retrouvée dans les jours ou semaines qui précédent les symptômes. Les anticorps quadrisyalylés anti GQ1b sont fortement positifs et présent chez plus de 90% des patients, car associés à une atteinte des fibres nerveuses oculo-motrices et rendant compte de l'ophtalmoplégie. Nous rapportons un cas de syndrome de Miller Fisher chez une patiente atteinte d'une infection pulmonaire à *Mycoplasma pneumonie* chez qui la recherche des anticorps anti GQ1b est négative.

## Patient et observation

Il s'agissait d'une femme de 75 ans, qui était venue en consultation pour des paresthésies à type de fourmillements des extrémités distales des deux membres supérieurs, et des sensations de percevoir au toucher tout objet comme du coton, le tout évoluant depuis 48 heures. L'interrogatoire retrouvait dans ses antécédents une bronchopathie chronique obstructive et un syndrome d'apnée du sommeil appareillé. La patiente recevait par ailleurs la Pristinamycine (Pyostacine^®^) prescrite par son médecin traitant depuis 10 jours pour un syndrome infectieux pulmonaire apparu une quinzaine de jours avant les paresthésies. A l'examen général, les paramètres vitaux étaient conservés, la patiente était apyrétique et elle avait une toux sans expectorations.

L'examen neurologique retrouvait une paralysie de la verticalité du regard plus marquée vers le haut, ressentie par la patiente depuis quelques jours, une aréflexie ostéo-tendineuse aux 4 membres. Il existait des troubles proprioceptifs marqués aux 4 membres associant une hypopallesthésie prédominant à gauche et plus sévères aux membres inférieurs, des troubles de la kinesthésie et un signe de Romberg présent, non latéralisé. Il n'y avait pas de trouble de l'oculomotricité intrinsèque, ni de nystagmus, ni de déficit moteur, ni de déficit de la sensibilité superficielle, ni de signes cérébelleux ou pyramidaux. L'auscultation pulmonaire mettait en évidence quelques discrets râles sous-crépitants diffus. Le reste de l'examen physique était sans particularité. Le syndrome de Miller Fisher était ainsi évoqué. Un électroneuromyogramme (ENMG) réalisé comportant une étude des vitesses de conduction sensitive et motrice, une étude des ondes F, une mesure des latences distales et des potentiels sensitifs et moteurs des nerfs des membres supérieurs et inférieurs ne retrouvait pas d'anomalie. La patiente était hospitalisée pour explorations complémentaires.

La radiographie pulmonaire ne trouvait pas de foyer parenchymateux évident. Le deuxième jour d'hospitalisation, le tableau clinique s'aggravait avec l'apparition d'une ophtalmoplégie complète et d'un discret ptosis gauche sans syndrome de Claude Bernard Horner. Le reste de la symptomatologie restait sans changement. Une ponction lombaire avec analyse du liquide cérébrospinal montrait une cellule nucléée et une protéinorachie à 0,40g/l. Une IRM cranio-encéphalique ne montrait aucune anomalie de signal au niveau du tronc cérébral. Un deuxième examen d'ENMG était superposable au précédent.

Les sérologies virales HIV, HTLV, Lyme et syphilis étaient négatives ainsi que la sérologie à *Campylobacter jejuni*. La sérologie *Chlamydiae sp*. était négative. La recherche des antigènes de légionellose était négative. La sérologie *Mycoplasma pneumoniae* était fortement positive (IgM à 1/320 pour une valeur référence inférieure à 1/40). Le diagnostic de syndrome de Miller Fisher était retenu. Les immunoglobulines intraveineuses à la dose de 400UI/kg/24 heures pendant 5 jours étaient administrées. La patiente était mise ensuite sous Télithromycine (Ketek^®^) par voie orale pendant 5 jours. L’évolution était marquée dès le 4^e^ jour de perfusion des Immunoglobulines polyvalentes par une amélioration clinique caractérisée par une reprise de la motilité oculaire latérale mais encore limitée. Deux semaines plus tard, l'on notait une récupération totale de la latéralité gauche et droite du regard et une récupération partielle de la verticalité aussi bien vers le haut que vers le bas. Les paresthésies avaient totalement disparues, les troubles de la sensibilité profonde étaient peu perceptibles, le signe de Romberg était absent et l'aréflexie ostéo-tendineuse était inchangée. La sérologie à *Mycoplasma pneumonie* contrôlée trois semaines plus tard était de 1/1280.

## Discussion

Le *Mycoplasma pneumoniae*, bactérie intracellulaire initialement décrite comme responsable des infections pulmonaires est aussi responsable des atteintes extra-pulmonaires parmi lesquelles les atteintes neurologiques. Les atteintes neurologiques sont centrales ou périphériques et consistent habituellement en une encéphalite ou méningo-encéphalite, les accidents vasculaires cérébraux ischémiques, les atteintes des nerfs crâniens, une myélite transverse, ou un syndrome de Guillain Barré.

Le diagnostic du syndrome du syndrome de Miller Fisher (SMF) est clinique et basé sur la triade classique: ophtalmoplégie, ataxie et aréflexie ostéo-tendineuse. Ces données cliniques sont confortées par des examens complémentaires: une hyperprotéinorrachie, la présence des Ac antiGQ1b dans le sérum et les anomalies électrophysiologiques. Il existe des formes extensives avec déficit moteur associé, cependant déficit moteur n’était associé dans notre cas.

Environ les 2/3 des patients atteints du SMF ont une hyperprotéinorrachie. Chez notre patiente, la protéinorrachie était normale. Six jours après le début des symptômes chez un patient atteint du SMF, Orr et Storey en 2004, notaient dans leur série que les vitesses de conduction sensitive et motrice ainsi que la latence de l'onde F étaient normales [10]. Les données de l’électroneuromyographie réalisée à deux reprises n'ont pas mis en évidence des signes de démyélinisation, ce qui n'est pas ne particularité car plusieurs auteurs ont également décrit des cas de SMF avec normalité de l'ENMG [2, 3]. L'ENMG n’était pas perturbée à la phase aiguë de l'affection chez cette patiente. Les vitesses de conduction étaient normales ainsi que les latences distales. Il n'existait pas d'anomalie évocatrice de démyélinisation, ni d'atteinte axonale. La normalité d'un ENMG n'exclut par conséquent pas le SMF si la clinique est classique.

La majorité des SMF surviennent après une infection respiratoire dans plus de 76% des cas [2, 4]. Le SMF a été rarement décrit comme étant associé à une infection à *Mycoplasma pneumoniae* [5–7].

Sur le plan immunologique, la recherche des AC antiGQ1b était négative chez notre patiente. Les anti-GM1 étaient faiblement positifs à 1/100^eme^. Les anti-GM2, GM3, GD3, GD1a, GD1b et GT étaient également négatifs. Chez plus de 90% des patients, les Ac anti GQ1b sont retrouvés dans le sérum à un titre élevé à la phase aigüe de la maladie. Le GQ1b est un tétrasyaloganglioside retrouvé au niveau de la surface cellulaire du système nerveux, mais très riche au niveau des noeuds de Ranvier des nerfs crâniens III, IV et VI. Le titre des anticorps est corrélé à la sévérité de l'ophtalmoplégie [2]. De même les anti-GD1b retrouvés chez certains patients étaient corrélés à l'ataxie sensitive [8].

Ces anticorps ne sont pas spécifiques du SMF car ils ont été également retrouvés dans le sérum des patients atteints du SGB et de l'encéphalite de Bickerstaff [9]. L'IRM réalisée chez notre patiente n'a pas mis en évidence des anomalies de signal dans la fosse postérieure. Un an après la description de Fisher en 1956, Bickerstaff a considéré le SMF comme étant en rapport avec une atteinte du tronc cérébral [10]. Il rapportait 8 cas d'encéphalite du tronc cérébral avec ataxie et surdité chez 6 patients, diminution ou abolition des réflexes ostéo-tendineux chez 5 patients. En 2003, Okada et al. à partir de 62 observations, décrivaient outre les signes décrits par Bickersatff, 74% des troubles de la conscience, une diplégie faciale dans 45% des cas, un signe de Babinski dans 40% des cas, une atteinte bulbaire dans 34% des cas, et un déficit moteur proximal dans 60% des cas [9].

Dans notre cas, tous ces anticorps étaient négatifs, même après une seconde recherche. Certains auteurs ont décrit des cas similaires de SMF avec absence d'Ac anti GQ1b [3]. L'absence des anticorps anti GQ1b dans le sérum de cette patiente à la phase aigüe de l'affection suggère l'existence d'un autre épitope que le GQ1b impliqué dans la pathogénie de cette affection.

## Conclusion

L'absence des anticoprs antiGQ1b ne doit pas faire exclure le diagnostic de SMF. La recherche d'une infection à *Mycoplasma pneumoniae* doit être systématique dans le bilan étiologique chez tout patient ayant les signes d'un syndrome de Miller Fisher.

## References

[1] Fisher M (1956). An unusual variant of acute idiopathic polyneuritis (syndrome of ophtalmoplegia, ataxia, and areflexia). N Engl J Med..

[2] Orr CF, Storey CE (2004). Recurrent Miller'Fisher syndrome. Journal of Clinical Neuroscience.

[3] Tan H, Caner I, Deniz O, Buyukavci M (2003). Miller Fisher syndrome with negative anti-GQ1b immunoglobulin G antibodies. Pediatr Neurol..

[4] Koga M, Gilbert M, Li J, Koike S, Furukawa K, Hirata K, Yuki N (2005). Antecedent infections in Fisher syndrome: a common pathogenesis of molecular mimicry. Neurology..

[5] Merkx H, Keyser (de) J, Ebinger G (1994). Miller Fisher syndrome associated with Mycoplasma pneumoniae infection: report of case. Clinical Neurology and Neurosurgery..

[6] Sanchez-Arjona BM, Macias EF, Villalobos chaves F (2003). Miller Fisher syndrome in the course of an pneumonia by Mycoplasma pneumoniae. Rev neurol..

[7] Hsueh KC, Chou IC, Hsu CH, Kuo HT, Tsai FJ, Tsai CH (2004). Miller Fisher syndrome possibly related to Mycoplasma pneumoniae infection: report of one case. Acta Peadiatr Taiwan..

[8] Dewarrat A, Regli F, Stek A, Kuntzer T (1995). Syndrome de Miller Fisher recurrent: signification des anticorps anti-GQ1b. Schweiz Arch neurol psychiatr..

[9] Odaka M, Yuki N, Yamada M, Koga M, Takemi T, Hirata K (2003). Bickerstaff's brainstem encephalitis: clinical features of 62 cases and a subgroup associated with Guillain Barre syndrome. Brain..

[10] Bickerstaff ER (1957). Brainstem encephalitis. Further observation on a grave syndrome with benign prognosis. BMJ..

